# Assessing implementation difficulties in tobacco use prevention and cessation counselling among dental providers

**DOI:** 10.1186/1748-5908-6-50

**Published:** 2011-05-26

**Authors:** Masamitsu Amemori, Susan Michie, Tellervo Korhonen, Heikki Murtomaa, Taru H Kinnunen

**Affiliations:** 1 Department of Oral Public Health, Institute of Dentistry, University of Helsinki, Helsinki, Finland; 2 Centre for Outcomes Research and Effectiveness, Department of Clinical, Educational and Health Psychology, University College London, London, UK; 3 Department of Public Health, Hjelt Institute, University of Helsinki, Helsinki, Finland; 4 Department of Oral Health Policy and Epidemiology, Harvard School of Dental Medicine, Harvard University, Boston, USA

## Abstract

**Background:**

Tobacco use adversely affects oral health. Clinical guidelines recommend that dental providers promote tobacco abstinence and provide patients who use tobacco with brief tobacco use cessation counselling. Research shows that these guidelines are seldom implemented, however. To improve guideline adherence and to develop effective interventions, it is essential to understand provider behaviour and challenges to implementation. This study aimed to develop a theoretically informed measure for assessing among dental providers implementation difficulties related to tobacco use prevention and cessation (TUPAC) counselling guidelines, to evaluate those difficulties among a sample of dental providers, and to investigate a possible underlying structure of applied theoretical domains.

**Methods:**

A 35-item questionnaire was developed based on key theoretical domains relevant to the implementation behaviours of healthcare providers. Specific items were drawn mostly from the literature on TUPAC counselling studies of healthcare providers. The data were collected from dentists (n = 73) and dental hygienists (n = 22) in 36 dental clinics in Finland using a web-based survey. Of 95 providers, 73 participated (76.8%). We used Cronbach's alpha to ascertain the internal consistency of the questionnaire. Mean domain scores were calculated to assess different aspects of implementation difficulties and exploratory factor analysis to assess the theoretical domain structure. The authors agreed on the labels assigned to the factors on the basis of their component domains and the broader behavioural and theoretical literature.

**Results:**

Internal consistency values for theoretical domains varied from 0.50 ('emotion') to 0.71 ('environmental context and resources'). The domain environmental context and resources had the lowest mean score (21.3%; 95% confidence interval [CI], 17.2 to 25.4) and was identified as a potential implementation difficulty. The domain emotion provided the highest mean score (60%; 95% CI, 55.0 to 65.0). Three factors were extracted that explain 70.8% of the variance: motivation (47.6% of variance, α = 0.86), capability (13.3% of variance, α = 0.83), and opportunity (10.0% of variance, α = 0.71).

**Conclusions:**

This study demonstrated a theoretically informed approach to identifying possible implementation difficulties in TUPAC counselling among dental providers. This approach provides a method for moving from diagnosing implementation difficulties to designing and evaluating interventions.

## Background

### Dental providers and tobacco use counselling

In addition to harmful effects on the respiratory and cardiovascular systems, tobacco use has significant adverse effects on oral health. Harmful effects vary from reduced ability to smell and taste to staining and discoloration of the teeth and dental restorations, implant failure, periodontal problems, and oral cancer [1-3]. In addition, evidence suggests a link between the dose-response effects of maternal tobacco use and orofacial clefts in infants [4]. Dental providers are in a key position to identify patients' tobacco use and to provide assistance in quitting once the first signs of tobacco use, such as bad breath and tooth discoloration, are evident. Therefore, dental consultations, usually done regularly and by the same person, provide an ideal opportunity for cessation counselling. Besides cessation, promoting tobacco abstinence is particularly important among young people who are about to experiment and initiate tobacco use. In Finnish community settings, dental providers meet about 75% of minors (< 18 years) each year [5], thus providing an excellent opportunity to promote abstinence. In addition, patients may welcome tobacco use prevention and cessation (TUPAC) counselling. Studies indicate that about 80% of tobacco users in Finland are worried about the harmful effects of smoking, and some 60% would like to quit [6]. Because dental visits provide an excellent platform for successful tobacco use intervention, the World Health Organization (WHO) Global Oral Health Programme has identified the implementation of TUPAC counselling guidelines as one of the priority goals in dentistry [7].

The Finnish Medical Society Duodecim has produced national Current Care guidelines for Smoking, Nicotine Addiction, and Interventions for Cessation. TUPAC counselling is based on what is known as the six *A*s approach [8], which is similar to the five *A*s used internationally [9]. The six *A*s approach recommends that healthcare providers ask about each patient's tobacco use at least once a year, assess and account for nicotine dependence and motivation to quit, advise patients to quit, assist them in quitting, and arrange for follow up.

Previous research has shown that dental providers are well aware of the harmful effects of tobacco use but often lack confidence in assisting patients to quit [10]. This lack of confidence may stem from lack of knowledge and skills, as well as from doubts about the effectiveness of TUPAC counselling, busy schedules, and lack of compensation [10-12], and has contributed to a widening gap between guideline recommendations and their implementation. Consequently, interventions designed to enhance dental providers' TUPAC guideline implementation are needed.

### Improving guideline implementation

The consensus report on TUPAC, the Second European Workshop on Tobacco Use Prevention and Cessation for Oral Health Professionals, proposed several ways to enhance TUPAC counselling among dental providers [13]. Recommendations included increasing undergraduate and continuing education on TUPAC counselling, as well as developing a TUPAC-related compensation system comparable to other therapeutic dental interventions. The evidence and theoretical basis for the effectiveness of such interventions are inconclusive, however, which highlights the need for more research on the implementation process and difficulties in guideline implementation.

There are many reasons for advocating a theory-based assessment of implementation problems. First, interventions are more likely to be effective if they target causal determinants of behaviour and behaviour change. Such targeting requires an understanding of theoretical mechanisms of change. Second, theory-based interventions facilitate an understanding of what works and thus creates a basis for developing a more accurate theory for different contexts, populations, and behaviours. Theoretical frameworks also provide a way of accumulating knowledge across empirical studies, thus creating a basis for developing more effective interventions. Growing recognition of these advantages has increased the demand for theory-based intervention evaluation to acquire data on behavior-change processes and critical factors involved in guideline implementation, which the UK's Medical Research Council (MRC) also advocated in their updated development and evaluation framework for complex interventions [14].

Although the MRC framework advocates the application of behavioural theory, it does not provide guidance as to how to do it. A plethora of theories of behaviour change abound, many of which share overlapping constructs, and none of which are comprehensive. A theoretical approach is needed that integrates such theories to extract a method to comprehensively assess implementation difficulties. A consensus group of health psychologists and implementation researchers has developed one such method. Based on their knowledge of behavioural and implementation theories, the group identified 12 key theoretical domains for investigating the implementation of evidence-based practice [15]. These domains are as follows: knowledge; skills; professional role and identity; beliefs about capabilities; beliefs about consequences; motivation and goals; memory, attention, and decision processes; environmental context and resources; social influences; emotion; behavioural regulation; and nature of the behaviours. An assessment based on these theoretical domains provides a basis for designing theory-based interventions that target those domains found to explain implementation difficulties. These domains have proved useful in implementation research [16-18]; however, simplifying and ordering them to provide a more parsimonious explanation of behaviour may provide an additional theoretical framework to inform future research.

### Aims and objectives

To improve our understanding of the difficulties dental providers face in implementing TUPAC counselling guidelines and to provide an evidence-based intervention design, this study aims to describe the development and use of a Theoretical Domain Questionnaire (TDQ). The objectives are to

• develop a TDQ for assessing implementation determinants of TUPAC guidelines among dental providers;

• apply the TDQ to a sample of dental providers to identify implementation difficulties;

• to uncover the possible underlying structure of the theoretical domains.

## Methods

### Development of the Theoretical Domain Questionnaire

To assess possible factors mediating the implementation of the TUPAC guidelines, we developed a questionnaire based on both the theoretical-domains framework [15] and the Finnish Current Care guidelines on TUPAC counselling [8]. The goal of the TDQ development was to measure each of the 12 domains, as well as the related key constructs within each domain.

First, we conducted a systematic literature review of published questionnaires on TUPAC counselling from PubMed using the following search terms: Topic = (tobacco OR smoking) AND Topic = (counselling OR counseling) AND Topic = (questionnaire OR survey) AND Topic = (dentist OR 'dental hygienist' OR hygienist OR nurse OR physician OR doctor OR 'healthcare provider' OR 'health care provider' OR 'general practitioner'). Of 1,240 articles (by 31 January 2009), we found about 60 different questionnaires that had served to assess the implementation of TUPAC guidelines among healthcare providers. Second, we contacted corresponding authors to request use of their questionnaire. Of the 25 questionnaires received, we found four questionnaires to be the most suitable, as they covered a wide range of implementation difficulties among healthcare providers [19-22]. Of these questionnaires, we assigned items under appropriate theoretical domains according to component constructs and elicited questions provided by the consensus group [15]. Because there were too few appropriate items for all domains, we created additional items (see Additional File 1). To maximise the chance that items reflect the main component constructs of each domain while keeping the questionnaire as short as possible, we sought the advice of experts on behaviour change and tobacco dependency treatment. The final version of the questionnaire consisted of 35 items (two to six items per domain) and covered the following 10 domains: knowledge; skills; professional role and identity; beliefs about capabilities; beliefs about consequences; motivation and goals; memory, attention, and decision processes; environmental context and resources; social influences; and emotion.

The questionnaire was developed in English, then translated and back-translated by independent translators (English-Finnish-English and English-Swedish-English) by Language Services, University of Helsinki. If differences between the original and the back-translated versions appeared, the questionnaire underwent a further round of back-translation until the versions showed satisfactory agreement. The questionnaire was piloted among a sample of dentists and dental hygienists (n = 30) working in municipal dental clinics in Helsinki, Finland. Piloting indicated that the providers understood and received the questionnaire well, and no changes were necessary.

We decided to exclude the domain behavioural regulation because in the context of community dental settings, the component constructs of behavioural regulation, such as goal/target setting, goal priority, feedback, project management, and barriers and facilitators [15], showed too much overlap with the domain environmental context and resources and were mediated mainly by the clinical environment and chief dental officers. Thus, this domain was considered less important that it would be in other settings, such as in private clinics. The domain nature of behaviour was also excluded, as it relates more to an understanding of the behaviour itself than to influences on behaviour [23]. A list of the domains, constructs, and items appear in Additional File 2.

### Participants

Dentists and dental hygienists employed by the municipal health centres of Vaasa (9 clinics) and Tampere (28 clinics), Finland, were invited to participate. To ensure the similarity of settings in all clinics, we excluded 3 of the 37 clinics. In Tampere, we excluded emergency and special treatment clinics, as well as the undergraduate education clinic in Vaasa. Participants meeting the inclusion criteria received an explanatory description of the study, a consent form, and instructions to participate [24]. The survey was conducted using either a web-based (http://www.surveymonkey.com) or more traditional paper form survey. Of the respondents, 98.6% (72/73) preferred the web-based survey. Strategies promoting response rates included offering two movie tickets (valued at about € 10 per ticket) for participation. Reminder letters were sent one week after the first request to respond, followed by another one sent to nonrespondents one week after the first reminder. The published study protocol [24] provides more detailed information on the participants, the exclusion criteria, and the setting.

### Statistical analysis

Estimates of internal consistency were calculated for the theoretical domains and factors using Cronbach's alpha, with a cutoff of 0.50, deemed sufficient for preliminary research [25]. Domain scores were based on responses measured on a five-point Likert scale (1 = strongly disagree, 5 = strongly agree) (Table 1); for negatively worded items, the scale scores were reversed. We calculated a total score for each domain and divided it by the maximum score for the given domain. The domain scores were reported as a percentage of the maximum possible. A low percent value suggests that that particular domain may be an area of difficulty for implementation, and a high percent value suggests that that particular domain may facilitate the implementation of guidelines. Correlation coefficients were calculated using Pearson's correlation and defined as low (0.0 to 0.39), moderate (0.40 to 0.69), or high (0.70 to 1.0).

**Table 1:** Internal consistency of domains (α) and the distribution of responses (1 = strongly disagree, 5 = strongly agree) among participants (n = 73)

KNOWLEDGE (α = 0.54)	1	2	3	4	5
I'm unaware of the meanings and objectives of the six *A*s in the Current Care guidelines on tobacco dependence treatment (Ask, Assess, Account, Advise, Assist, Arrange)*	7 (9.6)	12 (16.4)	25 (34.2)	15 (20.5)	14 (19.2)
I have sufficient therapeutic knowledge of the pharmaceutical products for tobacco cessation	26 (35.6)	27 (37.0)	12 (16.4)	7 (9.6)	1 (1.4)
I don't know how to promote a tobacco-free lifestyle among youth*	13 (17.8)	16 (21.9)	28 (38.4)	12 (16.4)	4 (5.5)

**SKILLS **(α = 0.55)	**1**	**2**	**3**	**4**	**5**

I know the appropriate questions to ask patients when providing tobacco use cessation counselling	28 (38.4)	23 (31.5)	17 (23.3)	3 (4.1)	2 (2.7)
I know how to prescribe pharmaceutical products for those ready to quit	34 (46.6)	20 (27.4)	9 (12.3)	8 (11.0)	2 (2.7)
I am unsure how to assess patients in their efforts to stop tobacco use*	2 (2.7)	8 (11.0)	23 (31.5)	18 (24.7)	22 (30.1)
Sufficient opportunities are available to learn about promoting a tobacco-free lifestyle	11 (15.1)	10 (13.7)	25 (34.2)	17 (23.3)	10 (13.7)

**PROFESSIONAL ROLE AND IDENTITY **(α = 0.57)	**1**	**2**	**3**	**4**	**5**

Most of my colleagues in this clinic believe that promoting tobacco abstinence is an important part of their professional identity	7 (9.6)	22 (30.1)	27 (37.0)	9 (12.3)	8 (11.0)
Counselling for cessation is not an efficient use of my time*	15 (20.5)	9 (12.3)	26 (35.6)	15 (20.5)	8 (11.0)

**BELIEFS ABOUT CAPABILITIES **(α = 0.64)	**1**	**2**	**3**	**4**	**5**

I am confident in my abilities to prevent patients from using tobacco products	17 (23.3)	25 (34.2)	26 (35.6)	2 (2.7)	3 (4.1)
I am able to make decisions about the risks/benefits of the appropriate use of nicotine replacement therapy	34 (46.6)	17 (23.3)	16 (21.9)	3 (4.1)	3 (4.1)
I have the skills to monitor and assist patients throughout their quit attempt	35 (47.9)	21 (28.8)	11 (15.1)	4 (5.5)	2 (2.7)

**BELIEFS ABOUT CONSEQUENCES **(α = 0.60)	**1**	**2**	**3**	**4**	**5**

My counselling will increase a patient's likelihood of quitting	7 (9.6)	18 (24.7)	24 (32.9)	21 (28.8)	3 (4.1)
Patients appreciate it when I promote tobacco abstinence	5 (6.8)	14 (19.2)	28 (38.4)	16 (21.9)	10 (13.7)
The patients we see in our clinic/department have so many other problems in their lives that stopping tobacco use is a very low priority for them*	3 (4.1)	14 (19.2)	27 (37.0)	20 (27.4)	9 (12.3)

**MOTIVATION AND GOALS **(α = 0.60)	**1**	**2**	**3**	**4**	**5**

I am unwilling to work on improving my provision of tobacco cessation services*	21 (28.8)	17 (23.3)	29 (39.7)	4 (5.5)	2 (2.7)
The importance of patient health helps me to overcome barriers such as lack of time and reimbursement in promoting a tobacco-free lifestyle	4 (5.5)	12 (16.4)	26 (35.6)	17 (23.3)	14 (19.2)
I receive insufficient reimbursement for promoting tobacco abstinence*	9 (12.3)	10 (13.7)	22 (30.1)	15 (20.5)	17 (23.3)
I have insufficient time to promote tobacco abstinence*	8 (11.0)	5 (6.8)	20 (27.4)	23 (31.5)	17 (23.3)

**MEMORY, ATTENTION, AND DECISION PROCESS **(α = 0.52)	**1**	**2**	**3**	**4**	**5**

Deciding whether to promote tobacco abstinence is sometimes difficult*	20 (27.4)	13 (17.8)	17 (23.3)	15 (20.5)	8 (11.0)
Reinforcing tobacco abstinence is easy for me to remember	8 (11.0)	14 (19.2)	23 (31.5)	19 (26.0)	9 (12.3)

**ENVIRONMENTAL CONTEXT AND RESOURCES **(α = 0.71)	**1**	**2**	**3**	**4**	**5**

My dental clinic has no tobacco-related self-help materials/pamphlets to distribute to patients*	5 (6.8)	8 (11.0)	9 (12.3)	10 (13.7)	41 (56.2)
Our dental clinic has a system to provide follow-up support between clinic visits	60 (82.2)	4 (5.5)	0	8 (11.0)	1 (1.4)
Our dental clinic has a system to cue/prompt providers to counsel against tobacco use	60 (82.2)	4 (5.5)	5 (6.8)	2 (2.7)	2 (2.7)
Our clinic management has taken actions to remove barriers to the provision of tobacco use counselling	27 (37.0)	8 (11.0)	23 (31.5)	12 (16.4)	3 (4.1)
In the dental clinic where I work, I receive no feedback from promoting tobacco abstinence*	1 (1.4)	7 (9.6)	16 (21.9)	11 (15.1)	38 (52.1)
My dental clinic provides insufficient reimbursement for promoting tobacco abstinence*	1 (1.4)	7 (9.6)	20 (27.4)	14 (19.2)	31 (42.5)

**SOCIAL INFLUENCES **(α = 0.52)	**1**	**2**	**3**	**4**	**5**

Our clinic/department generally supports improving the way in which we promote a tobacco-free lifestyle	16 (21.9)	10 (13.7)	28 (38.4)	13 (17.8)	6 (8.2)
Most patients do not want to receive tobacco counselling*	4 (5.5)	7 (9.6)	31 (42.5)	22 (30.1)	9 (12.3)
There is at least one respected individual in our dental clinic who is personally committed to leading our efforts to improve our provision of tobacco cessation services	44 (60.3)	10 (13.7)	11 (15.1)	4 (5.5)	4 (5.5)
My role does not involve assisting patients to stop tobacco use*	27 (37.0)	20 (27.4)	15 (20.5)	8 (11.0)	3 (4.1)
Most patients want to receive tobacco use cessation counselling	20 (27.4)	23 (31.5)	27 (37.0)	3 (4.1)	0

**EMOTION **(α = 0.50)	**1**	**2**	**3**	**4**	**5**

Helping with tobacco cessation makes me feel useful to patients	7 (9.6)	3 (4.1)	31 (42.5)	23 (31.5)	9 (12.3)
I find counselling patients about tobacco to be frustrating*	13 (17.8)	14 (19.2)	28 (38.4)	9 (12.3)	9 (12.3)
Burn-out prevents me from providing more tobacco use cessation counselling*	28 (38.4)	16 (21.9)	15 (20.5)	6 (8.2)	8 (11.0)

*Indicates negatively worded item, in which scales are reversed in further analysis.

We used the exploratory method for factor analysis because the theoretical-domain approach does not aim to identify causal processes of behaviour change per se, and no prior theory existed to explain behaviour change or behavior regulation. In factor analysis, theoretical domains served as the unit of analysis and met the conditions for exploratory factor analysis (Kaiser-Meyer-Olkin = 0.67, Bartlett's test < 0.001). For extraction criteria, we used an eigenvalue of 1.0 and the Varimax method for matrix rotation. The cutoff for factor loadings was set at 0.6, and statistical significance was set at *p *< .05. Factors were labelled based on their component domains and the broader behavioural and theoretical literature [23,26]. All analyses were performed using PASW Statistics version 18.0 (SPSS, Inc., Chicago, IL) for Mac OS X.

### Ethical review and study permissions

The Ethics Committees of the Pirkanmaa Hospital District and Vaasa Central Hospital approved our research plan, and the Research Permission Committee of the City of Tampere and the medical director of the Vaasa health centre granted us permission to conduct the study.

## Results

**Figure 1:**
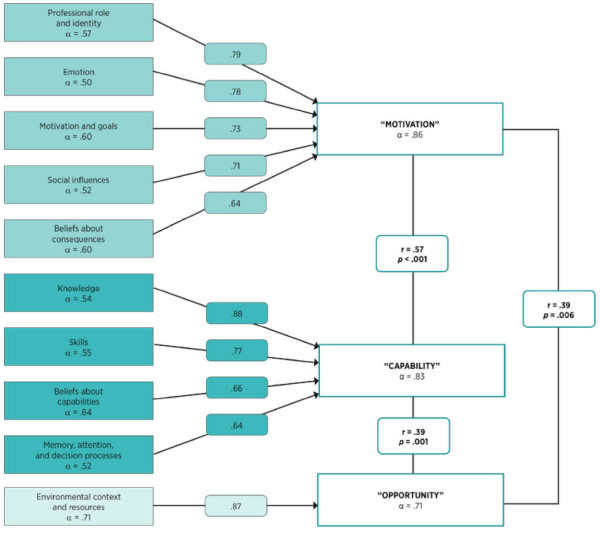
**Factors and theoretical domains with Cronbach's alpha (α) and domain loadings (> 0.60) (n = 73)**. Factor correlations (*r*) are provided with *p *values (two-tailed).

The response rate was 76.3% (73/95). Internal consistency for each theoretical domain was as follows: knowledge = 0.54; skills = 0.55; professional role and identity = 0.57; beliefs about capabilities = 0.64; beliefs about consequences = 0.60; motivation and goals = 0.60; memory, attention, and decision processes = 0.52; environmental context and resources = 0.71; social influences = 0.52; and emotion = 0.50 (Figure 1).

**Figure 2:**
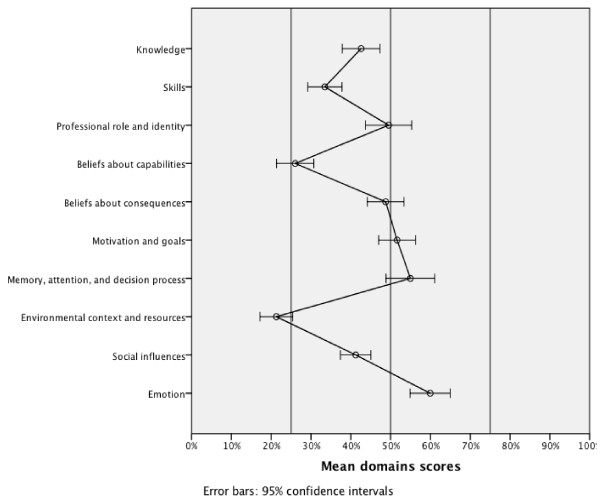
**The mean domain scores (total/maximum possible) with 95% confidence intervals (n = 73)**.

Reflecting the implementation difficulties, the mean scores (95% confidence interval [CI]) for the theoretical domains were as follows: knowledge = 42.6% (37.9 to 47.3); skills = 33.5% (29.2 to 37.8); professional role and identity = 49.5% (43.7 to 55.3); beliefs about capabilities = 26.0% (21.4 to 30.7); beliefs about consequences = 48.7% (44.1 to 53.4); motivation and goals = 51.6% (47.0 to 56.3); memory, attention, and decision processes = 55.0% (48.9 to 61.1); environmental context and resources = 21.3% (17.2 to 25.4); social influences = 41.2% (37.4 to 45.1); and emotion = 60% (55.0 to 65.0) (Figure 2). Correlations between domains were mostly low or moderate (Table 2). The domain motivation and goals correlated moderately with the following domains: professional role and identity (0.62; *p *< .001); social influences (0.57; *p *< .001); emotion (0.54; *p *< .001); memory, attention, and decision processes (0.44, *p *< .001); and beliefs about consequences (0.41; *p *< .001).

**Table 2:** Correlations between theoretical domains among dental providers (n = 73)

	Knowledge	Skills	Professional role	Capabilities	Consequences	Motivation	Memory and attention	Environmental resources	Social influences	Emotion
**Knowledge**	1									
**Skills**	0.60***	1								
**Professional role**	0.26*	0.39**	1							
**Capabilities**	0.50***	0.64***	0.51***	1						
**Consequences**	0.18	0.36**	0.53***	0.38**	1					
**Motivation**	0.31**	0.39**	0.62***	0.36**	0.41***	1				
**Memory and attention**	0.50***	0.47***	0.35**	0.42***	0.31**	0.44***	1			
**Environmental resources**	0.23	0.40***	0.19	0.28*	0.40**	0.34**	0.15	1		
**Social influences**	0.19	0.44***	0.59***	0.46***	0.71***	0.57***	0.35**	0.46***	1	
**Emotion**	0.20	0.41***	0.52***	0.42***	0.46***	0.54***	0.52***	0.22	0.52***	1

*p < .05; **p < .01; ***p < .001 (two-tailed).

Factor analysis of 10 domains yielded a three-factor solution, with a combined explained variation of 70.8% (Table 3). In considering the factor labels, we linked the work of other behavioural theorists, who conceptualised three factors necessary for behaviour to occur [20,23]. The factors were thus labelled as follows: motivation (47.6% of variance, α = 0.86), capability (13.3% of variance, α = 0.83), and opportunity (10.0% of variance, α = 0.71) (Table 2). Motivation consisted of five domains: professional role and identity, emotion, motivation and goals, social influences, and beliefs about consequences. Capability comprised the domains knowledge; skills; beliefs about capabilities; and memory, attention, and decision processes. Environmental context and resources comprised the third factor, opportunity. All correlations between factors were statistically significant (Figure 1).

**Table 3:** Rotated component matrix of theoretical domains and explained variance of each factor (n = 73)

DOMAINS	FACTORS		
	Motivation	Capability	Opportunity
**Knowledge**	0.033	0.88	0.083
**Skills**	0.24	0.77	0.35
**Professional role and identity**	0.79	0.21	0.11
**Beliefs about capabilities**	0.37	0.66	0.23
**Beliefs about consequences**	0.64	0.057	0.53
**Motivation and goals**	0.73	0.25	0.16
**Memory, attention and decision processes**	0.48	0.64	-0.15
**Social influences**	0.71	0.11	0.54
**Emotion**	0.78	0.26	-0.0020
**Environmental constraints**	0.086	0.21	0.87

**PERCENT OF VARIANCE**	47.6	13.3	10.0

Rotation method: Varimax with Kaiser normalisation.

## Discussion

### Main findings

This is one of the first quantitative and therefore testable reports applying a theoretical-domain framework to the task of identifying implementation difficulties of TUPAC counselling guidelines among dental providers. The results showed clear differences across theoretical domains, thus suggesting some explanations for implementation difficulties. The domains environmental context and resources, beliefs about capabilities, and skills yielded the lowest scores (Figure 2) and were thus identified as potential barriers to implementation. This result is consistent with findings from non-theory-based studies in other settings and contexts among dental providers [10-12,27]. Because the domain motivation and goals is potentially the most important predictor of guideline implementation among healthcare providers [16,28], it is encouraging that it produced a relatively high score in this context.

A recent review suggested that motivation and goals, beliefs about consequences, beliefs about capabilities, and social influences may play an important role in the behavior of healthcare providers [16]. In our study, motivation and goals was most highly (*r *> 0.50) associated with professional role and identity, social influences, and emotion (Table 2), whereas beliefs about consequences was associated with social influences and professional role and identity. Beliefs about capabilities proved to be most highly associated with skills and professional role and identity, and social influences was associated with beliefs about consequences, professional role and identity, motivation and goals, and emotion. Since professional role and identity, emotion, and skills were most highly associated with possible key domains [16], it seems that further analysis is needed to confirm our observations.

The internal consistency for the theoretical domains were in the acceptable range, from 0.50 (emotion) to 0.71 (environmental context and resources). From 10 theoretical domains, we extracted three factors. The first factor was labelled motivation, as the component domains all serve to energise (emotion and motivation and goals) and direct behavior (social influences, beliefs about consequences, and professional role and identity) (Table 3 and Figure 1). Component domains for the second factor, capability, are all aspects of physical or psychological capability and were thus named accordingly. The three factors, motivation, capability, and opportunity, have proved to be central constructs that explain behaviour [23] and closely represent Fishbein's intention, skills and abilities, and environmental factors [26].

Of the 10 domains, beliefs about consequences and social influences had impure factor loadings (>0.50 for two factors) (Table 3). As the domains beliefs about consequences and social influences include aspects that both motivate behaviour change and reflect environmental factors (Table 2 and Additional File 2), high loadings for both factors are understandable. And because extracting those two impure domains would have violated the construct of theoretical domains and reduced the explained total variance of factors, we decided to incorporate both domains in the analysis.

### Limitations

Although potentially useful, the framework approach does not identify the causal processes leading to behaviour change, per se. The theoretical-domain approach does not attempt to replace theories, but to identify barriers and provide relevant explanations for implementation difficulties. The TDQ cannot demonstrate all factors that contribute to the implementation of TUPAC guidelines among dental providers, since length constraints preclude measuring all aspects of each domain and select the key point of each. The allocation of certain items to domains was not always clear. For example, the item from the domain motivation and goals 'I have insufficient time to promote tobacco abstinence' could also be categorised as environmental context and resources. The rationale for our decision was that when taking time for certain operations, those deemed most important, for one reason or another, come first.

Excluding the domain behavioural regulation may have had some effect on the results of the factor analysis by emphasising the domain environmental context and resources, as the component constructs and items of these two did overlap. However, because other settings may depend more on behavioural regulation than does the current one, the present approach can be applied to a range of settings with possible differing domains.

It should be noted that the purpose of the current report was to develop and evaluate a questionnaire reflecting theoretical domains as behavioural determinants presumably related to TUPAC guideline-implementation behaviors in Finland. In TDQ development, we took into consideration a theoretical framework, published research in TUPAC, and TUPAC guidelines. Future examination and development are needed to evaluate how these domains relate to behaviours suggested in the TUPAC guidelines, such as the six *A*s, and how various interventions can change these behaviours.

### Implications

When designing interventions to enhance guideline implementation, target domains should be selected based on not only domain scores but also the relevance of each domain to behaviour change. Thus, intervention development should include identifying specific theories relevant to identified domains. For example, if the domain motivation and goals requires change, the Theory of Planned Behaviour may provide ideas for useful constructs to target (*e.g.*, attitude towards the behaviour or perceived control over a particular behaviour) and techniques relevant to changing those targets [29]. Social Cognitive Theory, on the other hand, may be useful for designing interventions to improve self-efficacy (beliefs about capabilities) [30]. In addition, specific interventions could be designed to address implementation difficulties based on theoretical domains. Because identified low self-efficacy (beliefs about capabilities) and skills may be potential barriers to implementation, strategies to enhance self-efficacy and skills rather than to focus solely on improving motivation (a high-scoring domain) could prove successful. Alternatively, strategies to develop and restructure the clinical environment (environmental context and resources) could be the best way forward.

In linking theoretical domains to behaviour-change techniques, one method could involve a matrix of domains mapped against 35 behaviour-change techniques [31]. Behaviour-change techniques such as problem solving, rehearsing relevant skills, and providing incentives could be selected according to relevant domains and target behaviours. These techniques may work best if designed for and adapted to the particular clinical context rather than rigidly standardised. However, our knowledge on selecting intervention techniques based on the theoretical assessment of implementation difficulties is at present limited and requires further research.

## Conclusion

This study has demonstrated a viable method to identifying implementation difficulties among dental providers using a theoretical-domains approach. The results provide a sound basis and starting point for designing interventions to improve the implementation of TUPAC counselling guidelines among dental providers.

## Competing interests

The authors declare that they have no competing interests.

## Authors' contributions

MA, TK, THK, and HM conceived the study and acquired funding. MA conducted the data analysis and wrote the first draft of the paper, as well as subsequent redrafts. SM and THK were theoretical and methodological advisers. All authors advised on clinical and methodological issues, provided ongoing critiques, and approved the final version of the manuscript.

## Supplementary Material

Additional file 1 **Theoretical domains, component constructs, and questionnaire items for investigating the implementation of tobacco use cessation counselling guidelines among dental providers**. Click here for file

Additional file 2 **Theoretical domains, component constructs, and questionnaire items for investigating the implementation of tobacco use cessation counselling guidelines among dental providers**. Click here for file
