# Supplementary material for: Assessing implementation difficulties in tobacco use prevention and cessation counselling among dental providers
Source: Implement Sci. 2011 May 26;6:50. doi: 10.1186/1748-5908-6-50 (PMC3130685; doi:10.1186/1748-5908-6-50)
Supplement: Additional file 1 — Theoretical domains, component constructs, and questionnaire items for investigating the implementation of tobacco use cessation counselling guidelines among dental providers. [file 1748-5908-6-50-S1.DOC]

| **Domain ITEMs** | **reference** |
| --- | --- |
| KNOWLEDGE |  |
| I’m unaware of the meanings and objectives of the Six A’s in the Current Care Guidelines on tobacco dependence treatment (ask, assess, account, advise, assist, arrange) | Modified from (19) |
| I have sufficient therapeutic knowledge of the pharmaceutical products for tobacco cessation | (20) |
| I don’t know how to promote a tobacco-free lifestyle among youth | New question |
| SKILLS |  |
| I know the appropriate questions to ask patients when providing tobacco use cessation counselling | (20) |
| I know how to prescribe pharmaceutical products for those ready to quit | New question |
| I am unsure how to assess patients in their efforts to stop tobacco use | Modified from (21) |
| Sufficient opportunities are available to learn about promoting a tobacco-free lifestyle | New question |
| PROFESSIONAL ROLE AND IDENTITY |  |
| Most of my colleagues in this clinic believe that promoting tobacco abstinence is an important part of their professional identity | Modified from (21) |
| Counselling for cessation is not an efficient use of my time | (20) |
| BELIEFS ABOUT CAPABILITIES |  |
| I am confident in my abilities to prevent patients from using tobacco products | Modified from (21) |
| I am able to make decisions about the risks/benefits of the appropriate use of nicotine replacement therapy | Modified from (21) |
| I have the skills to monitor and assist patients throughout their quit attempt | (20) |
| BELIEFS ABOUT CONSEQUENCES |  |
| My counselling will increase a patient’s likelihood of quitting | (20) |
| Patients appreciate it when I promote tobacco abstinence | Modified from (20) |
| The patients we see in our clinic/department have so many other problems in their lives that stopping tobacco use is a very low priority for them | (21) |

| MOTIVATION AND GOALS |  |
| --- | --- |
| I am unwilling to work on improving my provision of tobacco cessation services | Modified from (21) |
| The importance of patient health helps me to overcome barriers such as lack of time and reimbursement in promoting a tobacco-free lifestyle | Modified from (22) |
| I receive insufficient reimbursement for promoting tobacco abstinence | New question |
| I have insufficient time to promote tobacco abstinence | Modified from (20) |
| MEMORY, ATTENTION AND DECISION PROCESS |  |
| Deciding whether to promote tobacco abstinence is sometimes difficult | New question |
| Reinforcing tobacco abstinence is easy for me to remember | New question |
| ENVIRONMENTAL CONTEXT AND RESOURCES |  |
| My dental clinic has no tobacco-related self-help materials/pamphlets to distribute to patients | New question |
| Our dental clinic has a system to provide follow-up support between clinic visits | Modified from (21) |
| Our dental clinic has a system to cue/prompt providers to counsel against tobacco use | Modified from (21) |
| Our clinic management has taken actions to remove barriers to the provision of tobacco use counselling | New question |
| In the dental clinic where I work, I receive no feedback from promoting tobacco abstinence | New question |
| My dental clinic provides insufficient reimbursement for promoting tobacco abstinence | New question |
| SOCIAL INFLUENCES |  |
| Our clinic/department generally supports improving the way in which we promote a tobacco-free lifestyle | Modified from (21) |
| Most patients do not want to receive tobacco counselling | New question |
| There is at least one respected individual in our dental clinic who is personally committed to leading our efforts to improve our provision of tobacco cessation services | (21) |
| My role does not involve assisting patients to stop tobacco use | Modified from (21) |
| Most patients want to receive tobacco use cessation counselling | New question |

| EMOTION |  |
| --- | --- |
| Helping with tobacco cessation makes me feel useful to patients | (20) |
| I find counselling patients about tobacco to be frustrating | (20) |
| Burn-out prevents me from providing more tobacco use cessation counselling | New question |
