# Supplementary material for: Assessing implementation difficulties in tobacco use prevention and cessation counselling among dental providers
Source: Implement Sci. 2011 May 26;6:50. doi: 10.1186/1748-5908-6-50 (PMC3130685; doi:10.1186/1748-5908-6-50)
Supplement: Additional file 2 — Theoretical domains, component constructs, and questionnaire items for investigating the implementation of tobacco use cessation counselling guidelines among dental providers. [file 1748-5908-6-50-S2.DOC]

| **DOMAINS** | **CONSTRUCT** | **ITEMS IN QUESTIONNAIRE** |
| --- | --- | --- |
| KNOWLEDGE | Knowledge  Knowledge about condition/scientific rationale  Schemas+mindsets+illness representations  Procedural knowledge | I’m unaware of the meanings and objectives of the Six A’s in the Current Care Guidelines on tobacco dependence treatment (ask, assess, account, advise, assist, arrange)  I have sufficient therapeutic knowledge of the pharmaceutical products for tobacco cessation  I don’t know how to promote a tobacco-free lifestyle among youth |
| SKILLS | Skills  Competence/ability/skill assessment  Practice/skills development  Interpersonal skills  Coping strategies | I know the appropriate questions to ask patients when providing tobacco use cessation counselling  I know how to prescribe pharmaceutical products for those ready to quit  I am unsure how to assess patients in their efforts to stop tobacco use  Sufficient opportunities are available to learn about promoting a tobacco-free lifestyle |
| PROFESSIONAL ROLE AND IDENTITY | Identity  Professional identity/boundaries/role  Group/social identity  Social/group norms  Alienation/organisational commitment | Most of my colleagues in this clinic believe that promoting tobacco abstinence is an important part of their professional identity  Counselling for cessation is not an efficient use of my time |
| BELIEFS ABOUT CAPABILITIES | Self-efficacy  Control of behaviour and  social environment  Self-confidence/professional confidence  Empowerment  Self-esteem  Perceived behavioural control  Optimism/pessimism | I am confident in my abilities to prevent patients from using tobacco products  I am able to make decisions about the risks/benefits of the appropriate use of nicotine replacement therapy  I have the skills to monitor and assist patients throughout their quit attempt |
| BELIEFS ABOUT CONSEQUENCES | Outcome expectancies  Anticipated regret  Appraisal/evaluation/review  Consequents  Attitudes  Contingencies  Reinforcement/punishment/consequences  Incentives/rewards  Beliefs  Unrealistic optimism  Salient Events/sensitisation/critical incidents  Characteristics of outcome expectancies–physical, social, emotional;  Sanctions/rewards, proximal/distal,  valued/not valued, probable/improbable, salient/not salient,  perceived risk/threat | My counselling will increase a patient’s likelihood of quitting  Patients appreciate it when I promote tobacco abstinence  The patients we see in our clinic/department have so many other problems in their lives that stopping tobacco use is a very low priority for them |
| MOTIVATION AND GOALS | Intention; stability of intention/certainty of intention  Goals (autonomous, controlled)  Goal target/setting  Goal priority  Intrinsic motivation  Commitment  Distal and proximal goals  Transtheoretical model and stages of change | I am unwilling to work on improving my provision of tobacco cessation services  The importance of patient health helps me to overcome barriers such as lack of time and reimbursement in promoting a tobacco-free lifestyle  I receive insufficient reimbursement for promoting tobacco abstinence  I have insufficient time to promote tobacco abstinence |
| MEMORY, ATTENTION AND DECISION PROCESS | Memory  Attention  Attention control  Decision making | Deciding whether to promote tobacco abstinence is sometimes difficult  Reinforcing tobacco abstinence is easy for me to remember |
| ENVIRONMENTAL CONTEXT AND RESOURCES | Resources/material resources (availability and management)  Environmental stressors  Person x environment interaction  Knowledge of task environment | My dental clinic has no tobacco-related self-help materials/pamphlets to distribute to patients  Our dental clinic has a system to provide follow-up support between clinic visits  Our dental clinic has a system to cue/prompt providers to counsel against tobacco use  Our clinic management has taken actions to remove barriers to the provision of tobacco use counselling  In the dental clinic where I work, I receive no feedback from promoting tobacco abstinence  My dental clinic provides insufficient reimbursement for promoting tobacco abstinence |
| SOCIAL INFLUENCES | Social support  Social/group norms  Organisational development  Leadership  Team working  Group conformity  Organisational climate/culture  Social pressure  Power/hierarchy  Professional boundaries/roles  Management commitment  Supervision  Inter-group conflict  Champions  Social comparisons  Identity; group/social identity  Organisational commitment/alienation  Feedback  Conflict—competing demands, conflicting roles  Change management  Crew resource management  Negotiation  Social support: personal/professional/organisational, intra/interpersonal, society/community  Social/group norms: subjective, descriptive, injunctive norms  Learning and modelling | Our clinic/department generally supports improving the way in which we promote a tobacco-free lifestyle  Most patients do not want to receive tobacco counselling  There is at least one respected individual in our dental clinic who is personally committed to leading our efforts to improve our provision of tobacco cessation services  My role does not involve assisting patients to stop tobacco use  Most patients want to receive tobacco use cessation counselling |
| EMOTION | Affect  Stress  Anticipated regret  Fear  Burn-out  Cognitive overload/tiredness  Threat  Positive/negative affect  Anxiety/depression | Helping with tobacco cessation makes me feel useful to patients  I find counselling patients about tobacco to be frustrating  Burn-out prevents me from providing more tobacco use cessation counselling |
